# Food Addiction in a Group of Italian Adolescents Diagnosed for Eating Disorder

**DOI:** 10.3390/nu12051524

**Published:** 2020-05-23

**Authors:** Giulia Cinelli, Michela Criscuolo, Costanza Bifone, Ilenia Chianello, Maria Chiara Castiglioni, Antonino De Lorenzo, Laura Di Renzo, Alberto Eugenio Tozzi, Stefano Vicari, Valeria Zanna

**Affiliations:** 1 Predictive and Preventive Medicine Research Unit, Bambino Gesù Children Hospital IRCCS, 00165 Rome, Italy; giulia.cinelli@opbg.net (G.C.); albertoeugenio.tozzi@opbg.net (A.E.T.); 2 School of Specialization in Food Science, University of Rome Tor Vergata, 00133 Rome, Italy; 3 Demetra, Association for Eating Disorders’ Prevention and Care, 00198 Rome, Italy; michela_criscuolo@yahoo.it; 4 Anorexia Nervosa and Eating Disorder Unit, Child Neuropsychiatry, Dept. of Neuroscience, Bambino Gesù Children Hospital IRCCS, 00165 Rome Italy; costanza.bifone@gmail.com (C.B.); ilenia.chianello@opbg.net (I.C.); mariac.castiglioni@opbg.net (M.C.C.); 5 Clinical Nutrition and Nutrigenomic Section, Biomedicine and Prevention Department, University of Rome Tor Vergata, 00133 Rome, Italy; delorenzo@uniroma2.it (A.D.L.); laura.di.renzo@uniroma2.it (L.D.R.); 6 Department of Neuroscience, Child and Adolescent Psychiatric Unit, Bambino Gesù Children Hospital, IRCCS, 00165 Rome, Italy; stefano.vicari@opbg.net

**Keywords:** food addiction, adolescents, eating disorders, YFAS 2.0

## Abstract

Research in patients with Eating Disorders (EDs) showed high rates of Food Addiction (FA) even in restrictive subtypes. The majority of studies were conducted on adult population. The present work aimed to describe and compared FA in adolescents diagnosed for different EDs and to evaluate its association with patients’ psychopathology. Patients aged 12–18 y were included in the analysis. FA was assessed using the Yale Food Addiction Scale 2.0. The rate of FA was of 49.4% in the whole sample (*n* = 87, F = 90.8%) and of 53.7% in patients diagnosed with restrictive anorexia nervosa. No difference in FA frequency was detected between EDs. A worse psychopathological picture was found in patients diagnosed with FA. Higher age, higher score to the Eating Attitudes Test–26 and to the Eating Disorder Inventory-3′s Interoceptive Deficits scale have been detected as the major predictors of FA in our sample. FA may be considered a transnosographic construct, not linked to the subtype of ED but to patients’ personal characteristics and, in particular, to age and interoceptive deficits. A worse psychopathology might be considered a risk factor for the presence of FA in EDs.

## 1. Introduction

The concept of Food Addiction was firstly introduced in the 1950s by Theron Randolph (1956) in order to describe a dysregulated and dysfunctional eating behaviour typical of overweight and obese people [1]. Actually, it describes the excessive and uncontrolled consumption of high calorie and palatable foods, whose patterns are similar to those observed in substance-addicted patients. Therefore, FA has been defined as a “chronic and relapsing condition caused by the interaction of many complex variables that increase cravings for certain specific foods in order to achieve a state of high pleasure, energy or excitement, or to relieve negative emotional or physical states” [2].

In 2009, Geardhart et al. developed the Yale Food Addiction Scale (YFAS) to evaluate the presence of Food Addiction in patients with obesity or dysregulated eating pattern (Bulimia Nervosa (BN) and Binge Eating Disorder (BED)) [3,4,5]. Then, considering the DSM–5 (Diagnostic and Statistical Manual of Mental Disorders 5th Edition) significant changes to the substance-related and addictive disorders (SRAD), as, for example, the introduction of craving and severity levels, an updated version of the questionnaires, called YFAS 2.0, has been proposed [6]. Research showed that FA is about four times more frequent in subjects with an ED [7], particularly in those with BN [2], when compared to the general population [7,8]. Then, high rates of FA were also found in adult patients with Anorexia Nervosa, both restrictive and binge/purging [9,10]. However, the YFAS does appear to represent a distinct construct of problematic eating behaviour because not all patients who met the criteria for an ED exhibited YFAS-based food addiction [9].

Considering the DSM-5-based YFAS 2.0, a prevalence of FA of 3.4% was reported in Italy in a sample of 574 university students (mean age 21.4 y) [11]. This prevalence seems to be lower than those registered in other European or non-European countries, such as France (8.2%) [12], Germany (7.9%) [13] or United States (15.8%) [6], while it seems to be comparable to that found in other Latin countries, such as Brazil [14] or Spain (3.3%) [8], or in oriental countries such as Japan (3.3%) [15], suggesting a socio-cultural difference.

A small amount of studies has investigated FA in adolescents. Mies et al. found a prevalence of FA of 2.6% in a non-clinical sample of 2653 subjects aged 14–21 years in the Netherlands [16], while Zhao et al. detected a higher prevalence of 6.9% in China (mean age 15.0) [17]. Recently, Albayrak et al. conducted a study on adolescent psychiatric inpatients (13–18 years), finding that ED patients with a comorbidity of FA were more prone to present more severe clinical and psychopathological conditions, such as emotional dysregulation, low self-esteem and a major risk of dysfunctional eating-pattern chronicity when compared to those without FA [9]. All these studies used the old DSM-IV-based version of YFAS.

At present, the majority of studies in Italy were conducted on adult non-clinical subjects in order to validate or to assess the psychometric properties of the different version of the YFAS (YFAS, YFAS 2.0, modified YFAS 2.0) [11,18,19]. Therefore, there is a lack of knowledge about the presence of FA and its symptoms in clinical and adolescent groups, such as those with EDs [19]. Therefore, it could be relevant to investigate the presence of a food addiction symptomatology in this population in order to explore the possible association with a specific ED. In particular, since the comorbidity between FA and other EDs is associated with worse clinical conditions and symptoms [9,20] it is of interest to better understand if it is possible to highlight early indicators in the food pattern or psychopathological symptomatology, to then intervene at a therapeutic level.

In light of the literature evidence, the main objective of the following study was to describe the presence and severity of FA in a group of adolescents diagnosed with an ED using the latest version of the scale (YFAS 2.0). Secondly, we aimed to (i) compare FA frequency and severity in the different subgroups of ED; (ii) investigate the symptom count in the different subgroups of EDs and (iii) evaluate the association between FA and patients’ psychopathology.

## 2. Materials and Methods

### 2.1. Subjects and Study Design

The data of patients admitted to the Neuropsychiatry Unit of the Bambino Gesù Children Hospital and diagnosed with Eating Disorders from March 2018 and May 2019 were collected for the present cross-sectional study. Inclusion criteria were: male and female, age between 12 and 18 years and primary diagnosis of ED based on DSM-5 criteria. Patients with intellectual disabilities, substance abuse and a non-ED primary diagnosis were excluded.

All the patients included in the present study were admitted to the Hospital to undergo a first evaluation for the diagnosis. The assessment protocol was configured over three days and includes the following: anamnesis, nutritional assessment, psychological assessment (psychiatric interview, interview for family diagnosis, psychometrics) [21]. The diagnostic assessment was made at the moment of referral to the inpatient unit by a trained psychiatrist (V.Z.), who first made the diagnosis through a routine clinical interview and then used the Italian version of the Schedule for Affective Disorders and Schizophrenia for School-Age Children/Present and Lifetime Version (K-SADS-PL DSM- 5) [22] to confirm it as well as psychiatric comorbidity.

All the participants and their parents, when admitted to the Hospital, signed a consent for the use of their clinical data for research purposes.

### 2.2. Psychometric Measures

During the clinical assessment, each patient received a package containing the psychometric battery of self-administered questionnaires and was asked to complete them. Later, psychologists performed the scoring of all the questionnaires. Emotional, behavioural characteristics and psychopathological dimensions were assessed with: the Multidimensional Anxiety Scale for Children 2 (MASC 2) [23,24], the Children Depression Inventory 2 (CDI 2) [25,26] and the Youth Self-Report (YSR) [27,28]. Eating disorder psychopathology, food addiction and the possible presence of dysmorphophobia were investigated using the Eating Disorder Inventory-3 (EDI-3) [29,30], the Eating Attitudes Test–26 (EAT-26) [31], the Yale Food Addiction Scale 2.0 (YFAS-2.0) [11] and the Body Uneasiness Test (BUT) [32], respectively.

#### 2.2.1. Multidimensional Anxiety Scale for Children 2

The Multidimensional Anxiety Scale for Children 2 (MASC 2) [23] is a questionnaire for the evaluation of the main dimensions of anxiety in children and adolescents from 8 to 19 years of age. The self-report form contains 50 items, which measure: Separation/Fears, Generalized Anxiety (GAD) Index, Obsessions/Compulsions, Harm Avoidance, Social Anxiety (Humiliation/Rejection and Performance Fears) and Physical Symptoms (Panic and Tense/Restless). The Italian version of MASC 2 has shown excellent validity, like the original version, a good internal consistency and test-retest reliability [24].

#### 2.2.2. Children Depression Inventory 2

The Children Depression Inventory 2 (CDI 2) [25] is a self-report questionnaire for the evaluation of the depressive symptoms of children and adolescents from 7 to 17 years of age. It is made up of sets of items, each containing three options that reflect the severity of the symptom, from 0 (absent) to 2 (defined, marked). From self-report form, clinicians get a Total Score and scores on two scales: Emotional Problems and Functional Problems. In addition, it provides scores for four further sub-scales, called Negative Mood/Physical Symptoms, Negative Self-Esteem, Ineffectiveness and Interpersonal Problems. The set of statistical surveys highlighted the quality of the test items, its reliability and its validity in the Italian version [26].

#### 2.2.3. Youth Self-Report

To assess the adolescents’ view of their behaviour and socioemotional functioning, the Italian version of the Youth Self-Report (YSR) [26] was used. This questionnaire has to be completed by the 11- to 18-year-old adolescent and contains 112 problem items, covering behavioural, emotional, and social problems that occurred during the past 6 months. The YSR can be scored on syndrome scales: Anxious/Depressed, Withdrawn/Depressed, Somatic Complaints, Social Problems, Thought Problems, Attention Problems, Aggressive Behaviour, and Rule-Breaking Behaviour. The Internalizing scale can be derived from the first three syndrome scales, and the Externalizing scale from the last two. This measure, in its validated Italian version, has demonstrated very good day test–retest reliability, cross-informant agreement, and success in discriminating between referred and no referred adolescents [27].

#### 2.2.4. Eating Disorder Inventory-3

The Eating Disorder Inventory-3 (EDI-3) [28] is a self-report instrument measuring psychological traits or constructs shown to be clinically relevant in individuals with ED. This measure consists of 91 items organized into 12 primary scales, three eating disorder-specific scales (Drive for Thinness—DT; Bulimia—B; Body Dissatisfaction—BD) and nine general psychological scales (Low Self-Esteem—LSE; Personal Alienation—PA; Interpersonal Insecurity—II; Interpersonal Alienation—IA; Interoceptive Deficits—ID; Emotional Dysregulation—ED; Perfectionism—P; Asceticism—A; Maturity Fears—MF) that are highly relevant to, but not specific to, eating disorders. The reliability coefficients of the scales range from 0.83 and 0.90, and test–retest reliability coefficients for the various composite scales are between 0.84 and 0.87. The Italian version of EDI-3 [29] has demonstrated very good day test–retest reliability, cross-informant agreement, and a good discriminating validity.

#### 2.2.5. Eating Attitudes Test–26

The Italian version of the Eating Attitudes Test–26 [30] contains 26 items that assess the characteristics and symptoms of eating disorders and has been validated as both a dimensional and categorical measure. Individuals who score 20 or higher on this measure are considered to be at risk of, or likely to have clinical levels of, eating disorder symptoms. Participants indicate the extent to which they endorse each item along a 6-point scale that ranges from 1 (never) to 6 (always), with higher scores reflective of more severe symptomatology.

#### 2.2.6. Yale Food Addiction Scale 2.0

The Italian version of the Yale Food Addiction Scale 2.0 (YFAS-2.0) is a self-reported questionnaire used to assess addiction-like eating behaviour [11]. Each of the 35 question is referred to the past 12 months and falls under a DSM-5 SRAD symptom criterion or clinical impairment/distress. They are scored on an eight-point scale ranging from 0 (never) to 7 (every day) that account for 11 symptoms. There is no sum score calculated from the single items of the YFAS-2.0; each of the 11 diagnostic criteria is considered fulfilled if one or more of the relevant questions for each criterion reached the threshold. The YFAS 2.0 symptom count is calculated as the sum of the number of fulfilled diagnostic criteria (ranging from 0 to 11). FA diagnosis, instead, requires the presence of the impairment/distress criteria. Hence, for the “diagnosis” scoring option, both the symptom count score and the clinical significance criterion are used:

No Food Addiction = 1 or fewer symptoms/Does not meet criteria for impairment/distress criteria;
Mild Food Addiction = 2 or 3 symptoms and impairment/distress criteria;
Moderate Food Addiction = 4 or 5 symptoms and impairment/distress criteria;
Severe Food Addiction = 6 or more symptoms and impairment/distress criteria.

#### 2.2.7. Body Uneasiness Test

The Body Uneasiness Test [31] was used for the clinical assessment of body uneasiness. The BUT-A consists of four subscales and a global severity index (GSI) that have been demonstrated to have good internal consistency and reliability: Weight Phobia (WP—fear of being or becoming fat), Body Image Concerns (BIC—worries related to physical appearance), Avoidance (A—body image-related avoidance behaviour), Compulsive Self-Monitoring (CSM—compulsive checking of physical appearance), and Depersonalization (D—detachment and estrangement feelings toward the body). The Italian version of the instrument shows good reliability coefficients and a factorial structure congruent with the operative definition of the construct [31].

### 2.3. Statistical Analysis

Data are represented as number and percentage in parentheses (%) for categorical variables, or median and interquartile range in square brackets [IQR] for continuous variables. The Shapiro-Wilk test was performed in order to evaluate variables distribution. All the variables had skewed distribution.

Mann–Whitney U and Kruskal–Wallis tests were performed in order to compare patients’ characteristics (age and BMI) and psychometrics tests’ scores (YSR, CDI 2, MASC 2, EAT-26, BUT, EDI-3) among two or more groups (FA absent/present or FA mild/moderate/severe), respectively. Mann–Whitney U analysis was also used to investigate the difference between YFAS 2.0 symptom count between pairs of EDs. The Spearman correlation coefficient, instead, was calculated in order to evaluate the correlation between YFAS 2.0 symptom count and BMI. The exact Fisher test was used to evaluate the association between the presence of FA, FA severity, FA’s symptoms and categorical variables (ED diagnosis).

Univariable logistic regression analyses were conducted to investigate the association between FA (the dependent variable) and psychometric scales of YSR, CDI 2, MASC 2, EAT-26, BUT and EDI-3 (independent variables). Then, the association among FA and psychometric scales was further explored by developing a multivariable logistic regression model, while adjusting for patients’ age, considering only variables significantly associated to FA (*p* < 0.2 at univariable analysis). The final multivariable models were determined through a backward approach.

Results were significant for *p*-value < 0.05.

Statistical analysis was performed through IBM SPSS Statistics V21.0.

### 2.4. Ethics Approval

The use of clinical data for the present research was approved by the Ethical Committees of the Bambino Gesù Children Hospital (protocol number 1909_OPBG_2019) in full agreement with the national and international regulations and the Declaration of Helsinki (2000).

## 3. Results

### 3.1. Subjects

From the initial cohort of 101 patients aged 12–18 years and diagnosed for ED, 12 were excluded for a non-ED primary diagnosis while two were excluded for substance abuse. Finally, 87 patients were included in the analysis for the present study: nine met the DSM-5 criteria for avoidant restrictive food intake disorder (ARFID, 10,3%), forty-one for restrictive anorexia nervosa (R-AN, 47,1%), seven for bulimia nervosa (BN, 8,0%), two for binge eating disorder (BED, 2,3%), eight for eating disorder not otherwise specified (ED-NOS, 9,2%), one for binge purging anorexia nervosa (BP-AN, 1,1%), 19 for atypical anorexia nervosa A-AN (21,8%). Subjects’ characteristics are shown in Table 1.

### 3.2. FA in the Different Subgroups of EDs

Considering the whole sample, 43 patients (49.4%) met the criteria for the diagnosis of FA, of which 15 (17.2%), 12 (13.8%) and 16 (18.4%) were diagnosed with mild, moderate and severe FA, respectively. We found no significant difference for the presence of FA, as well as for its severity, when considering the different diagnosis of EDs. No difference was found, even excluding BED and AN-BP, which were the less representative groups. A significant result was, instead, found for the YFAS 2.0 symptoms count, which appear to be different among the ED groups: BED had a higher score when compared to ARFID (Mann–Whitney U = 18.00, *p* = 0.036), R-AN (Mann–Whitney U = 80.50, *p* = 0.004), ED-NOS (Mann–Whitney U = 0.00, *p* = 0.044) and A-AN (Mann–Whitney U = 1.50, *p* = 0.019). Specifically the “amount” and “craving” symptoms were found to be significantly more frequent in BN and BED.

**Figure 1:**
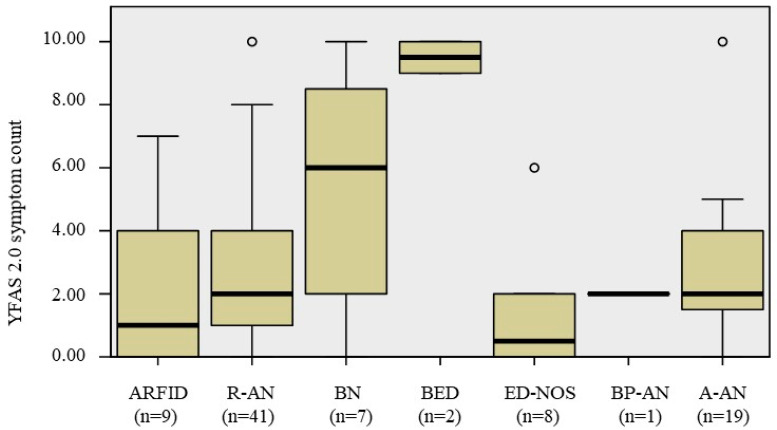
Boxplot for YFAS 2.0 symptom count. The symptom count includes the 11 symptoms without the “impairment/distress”. The Shapiro–Wilk test showed a non-normal distribution of the variable (*p* = 0.000). A-AN, atypical anorexia nervosa; ARFID, avoidant restrictive food intake disorder; BED, binge eating disorder; BN, bulimia nervosa; BP-AN, binge purging anorexia nervosa; ED-NOS, eating disorder not otherwise specified; R-AN, restrictive anorexia nervosa; YFAS, Yale food addiction scale.

Table 2 includes the distribution of FA and its symptoms in the different Eds, while Figure 1 shows the boxplots for YFAS 2.0 symptom count.

### 3.3. FA and Patients’ Characteristics

When considering the diagnosis of FA no difference was found in BMI percentile (*p* = 0.334) unlike the age distribution: patients with FA were older than those without (Mann–Whitney U = 1204.50, *p* = 0.028).

On the contrary, no difference in age distribution was found when considering FA severity (mild, moderate, severe) (*p* = 0.627), while BMI percentile resulted was found to be higher in severe FA compared to mild FA (*p* = 0.022). Moreover, the YFAS 2.0 symptom count was positively correlated with BMI percentile (r = 0.275, *p* = 0.010).

### 3.4. FA and Psychopathology and Eating Pattern

YSR, MASC 2 and CDI 2 were used to assess patients’ behavioural characteristics and psychopathological dimensions, while EAT-26, BUT and EDI-3 were used to investigate eating pattern and psychopathology and body satisfaction. Statistically significant higher tests scores were found in patients with FA when compared to those without (Supplementary Materials Table S1). We further investigate the difference in psychometric tests scores among the three severity levels of FA. No significant difference was found (Supplementary Materials Table S2). The results from univariable logistic regression show higher scores at psychometric tests, except for BUT, being statistically associated with an increased risk of FA. A total of 79 patients, for which all the test scales were available, was then included in the multivariable logistic regression; 38 (48.1%) were diagnosed with FA. The final step of the backward approach shows an association between the presence of FA and higher age, higher scores to EAT-26 and to the EDI-3′s Interoceptive Deficits scale (Table 3). Supplementary Materials Table S3 shows scores and univariable logistic regression results for the psychometric tests included in the multivariable model.

## 4. Discussion

Our results show a frequency of FA in adolescent ED patients of 49.4%. The overall rate of FA is comparable to that found by a previous study by Albayrak et al. which examined 37 inpatients adolescent (13–18 years) diagnosed with ED, with a frequency of FA of 45.9% [9]. They used an old version of the YFAS [3] in a smaller sample *n* = 37. On the contrary, our result is lower than that found by Granero et al. in a sample of 135 adult patients with ED (mean age 31.4), 77.8% of which were diagnosed with FA using the YFAS 2.0 [8]. This discrepancy is probably due the difference in samples’ age; in fact, we found FA to be positively associated with this variable.

When compared to the non-clinical adolescent population our results confirm, as expected, the higher prevalence of FA in the presence of psychiatric comorbidity, such as patients with ED [9]. In their sample of 2653 subjects aged 14–21 years, Mies et al. found 2.6% of them meeting the criteria for the diagnosis of FA using the YFAS for children [16]. Instead, Zhao et al. reported that 6.9% of their 13–17 year-old sample (*n* = 593) were found to be food-addicted based on the first version of the YFAS. Differently to our results, the latter study found no significant differences in age between the two groups with or without FA [17]. Probably, non-clinical populations show a more constant eating pattern over time compared to patients with an ED, in which the evolution of the disorder may lead to uncontrolled eating behaviours. In our research, we found no correlation between BMI percentile and diagnosis of FA. These results are similar to those detected by Albayrak et al. in their entire sample (242 psychiatric inpatient adolescents), but in contrast to what was reported by previous research focused on non-clinical, overweight and obese adolescents [9,17,33] or adult patients with BN and BED [20]. Likely, it is due to the high frequency of FA found in our subgroups of AN patients. Nevertheless, we detected a positive correlation between BMI percentile and the YFAS 2.0 symptom count. As expected, in fact, BN and BED patients had a higher BMI percentile and higher symptom count.

Interestingly, we found no difference in the diagnosis of FA when comparing the subgroups of EDs, even when excluding BED and BP-AN, which were the less representative groups. Moreover, we investigated this difference between R-AN and BN or A-AN, finding no statistically significant results. This is again in line with what was already detected by Albayrak et al., who found no difference when comparing the frequency of FA in 28 adolescent patients with AN and eight with BN [9]. On the contrary, a difference was found in the symptom count, with higher scores found in BED patients. In particular, for the scales “amount” and “craving”, a higher frequency was found in BN and BED.

In our study sample, more than half of R-AN patients (53.7%) were found to be food-addicted. Granero et al., 2014, found similar rates in adults with AN restrictive subtype (50%), while a lower rate was found by Albayrak et al. (42.8%) in their sample of 28 adolescents with R-AN [9,10].

The reason that patients with a restrictive eating pattern, who generally avoid high-caloric and high-palatable foods, met the criteria for FA is not yet clear. Previous studies speculate about how AN patients could interpret YFAS questions and on the fact that the answers could be driven by their perceived impulses to food rather than actual intake [9]. Often, restrictive eating pattern reflects the extreme effort of patients to fight against an internal impulse towards food or the pleasure derived from it. In fact, not rarely, patients with AN are afraid of not being able to stop eating the foods they like most. In other cases, even if AN patients generally avoid any food associated with the possibility of gaining weight, they are not able to stop eating a small quantity of some food that they are “addicted” to [11]. This could explain the “withdrawal” symptom, found to be positive in almost a half of our R-AN patients (44%). Our hypothesis is that the diagnosis of FA in R-AN could be a predictor of the shift to a binge eating behaviour, which may occur in some but not all restrictive patients. In support of this, a previous study by Castellini et al. detected an association between the crossover from AN to BN diagnosis and the presence of substance abuse [34].

Finally, concerning psychopathology, we found a higher score in psychometric tests investigating depression, anxiety and dysfunctional eating pattern, confirming that patients with FA show a worse psychopathological picture [20]. In particular, higher age, and a higher score in EAT-26 and the EDI-3′s Interoceptive Deficits scale have been detected as the major predictors of FA in our sample. The Interoceptive Deficits scale investigates confusion in recognition of and response to emotional states. It is coherent that a high score on this scale corresponds to a greater risk of FA, that is a greater use of food as a behavioural response to an emotional difficulty that the patient does not recognize or cannot cope with. This in line with the existing literature: BN and BED patients, which have uncontrolled eating patterns, have been shown to get higher scores on this scale [35,36].

The main limitations of the present study are the small sample size and, in particular, the difference between ED groups’ sizes. Therefore, our population may not be representative of universal adolescent EDs and our results may not be generalizable.

## 5. Conclusions

To our knowledge, this is the first study investigating the presence and severity of FA in a group of outpatient adolescents diagnosed for ED by using the new version of the Yale Food Addiction Scale (YFAS 2.0) and psychometric tools for the assessment of the related psychopathological risk. Overall, this study corroborates and extends the results of previous research. Our findings suggest that FA may be considered a transnosographic construct, not linked to the subtype of ED but to patients’ personal characteristics, and in particular to age and interoceptive deficits. A worse psychopathology, as well as dysfunctional eating pattern, are associated with the diagnosis of FA and might be considered risk factors for this construct in patients with an ED. Hence, it would be necessary to consider the presence of FA during the assessment and treatment of EDs in adolescent patients.

As one of the limitations of the study is the small sample size of BP-AN and BED patients, additional investigations are needed in this field. Finally, since restrictive EDs have shown a higher rate of FA, further research is needed in order to better understand this eating pattern in larger samples of adolescent patients diagnosed with R-AN, and to investigate its role as a predictor of the switch to binge subtypes, such as BP-AN, BN or BED.

## Figures and Tables

**Table 1:** Patients’ characteristics.

	Whole Sample (*n* = 87)	ARFID (*n* = 9)	R-AN (*n* = 41)	BN (*n* = 7)	BED (*n* = 2)	ED-NOS (*n* = 8)	BP-AN (*n* = 1)	A-AN (*n* = 19)	*p* *
Age	15.60 (2.8)	14.10 (3.9)	16.00 (2.40)	16.30 (1.80)	14.75 (-)	16.10 (3.05)	16.60	15.10 (3.20)	0.000
Gender (F)	79 (90.8)	5 (55.6)	41 (100.0)	7 (100.0)	1 (50.0)	6 (75.0)	1 (100.0)	18 (94.7)	-
Weight (kg)	43.50 (11.80)	38.60 (11.90)	41.60 (8.40)	54.20 (2.80)	116.20 (-)	38.9 (18.2)	41.30	49.10 (11.60)	**0.003**
Height (cm)	159.00 (9.50)	160.50 (13.50)	159.00 (7.50)	157.00 (9.80)	166.50 (-)	157.00 (6.50)	160.00	159.00 (11.0)	0.259
BMI (kg/cm^2^) ^a^	17.24 (3.55)	14.98 (2.74)	16.47 (2.87)	21.58 (2.30)	41.95 (-)	15.81 (4.07)	16.13	19.43 (2.61)	**0.000**
BMI percentile ^a^	10.00 (32.00)	3.00 (15.5)	4.0 (9.5)	65.00 (43.0)	99.0 (-)	5.00 (16.3)	1.0	40.00 (34.00)	**0.000**

Values are expressed as median and IQR (M [IQR]) for continuous variables or as number and percentage (*n* (%)) for categorical variables. ^a^ For BED and BP-AN the IQR has not been calculated as the groups included two and one subjects, respectively. * The Shapiro–Wilk test was performed to evaluate variables distribution. Variables are considered non-normally distributed for *p* < 0.05 (in bold). A-AN, atypical anorexia nervosa; ARFID, avoidant restrictive food intake disorder; BED, binge eating disorder; BMI, Body Mass Index; BN, bulimia nervosa; BP-AN, binge purging anorexia nervosa; ED-NOS, eating disorder not otherwise specified; EDs, eating disorders, R-AN, restrictive anorexia nervosa; YFAS, Yale food addiction scale.

**Table 2:** Food Addiction in the different subgroups of Eating Disorders.

	Whole Sample (*n* = 87)	ARFID (*n* = 9)	R-AN (*n* = 41)	BN (*n* = 7)	BED (*n* = 2)	ED-NOS (*n* = 8)	BP-AN (*n* = 1)	A-AN (*n* = 19)	Test Value	*p*	V di Cramer ^a^
**Food Addiction**											
Positive	43 (49.4)	3 (33.3)	22 (53.7)	4 (57.1)	0 (0)	3 (37.5)	1 (100)	10 (52.6)	4.520	0.653 ^a^	-
**Severity**											
Mild	15 (17.2)	1 (11,1)	8 (19,5)	0 (0)	0 (0)	2 (25)	1 (100)	3 (15,8)	9.630	0.416 ^a^	-
Moderate	12 (13.8)	1 (11,1)	7 (17,1)	0 (0)	0 (0)	0 (0)	0 (0)	4 (21,1)	-	-	-
Severe	16 (18.4)	1 (11,1)	7 (17,1)	4 (57,1)	0 (0)	1 (12,5)	0 (0)	3 (15,8)	-	-	-
**Yfas 2.0 Symptoms**											
Amount	16 (18,40)	1 (11.1)	4 (9.8)	5 (71.4)	2 (100.0)	1 (12.5)	0 (0.0)	3 (15.8)	18.425	**0.001** ^a^	0.543
Attemps	22 (25,30)	1 (11.1)	12 (29.3)	2 (28.6)	2 (100.0)	1 (12.5)	0 (0.0)	4 (21.1)	6.862	0.300 ^a^	--
Time	35 (40,20)	4 (44.4)	14 (34.1)	4 (57.1)	2 (100.0)	2 (25.0)	1 (100.0)	8 (42.1)	6.311	0.367 ^a^	--
Activities	27 (31,00)	2 (22.2)	15 (36.6)	4 (57.1)	0 (0.0)	0 (0.0)	0 (0.0)	6 (31.6)	7.676	0.227 ^a^	--
Consequences	24 (27,60)	2 (22.2)	12 (29.3)	4 (57.1)	2 (100.0)	1 (12.5)	0 (0.0)	3 (15.8)	9.545	0.101 ^a^	--
Tolerance	21 (24,10)	2 (22.2)	8 (19.5)	3 (42.9)	2 (100.0)	1 (12.5)	0 (0.0)	5 (26.3)	7.690	0.217 ^a^	--
Withdrawal	44 (50,60)	4 (44.4)	18 (43.9)	5 (71.4)	2 (100.0)	3 (37.5)	0 (0.0)	12 (63.2)	6.325	0.363 ^a^	--
Problems	28 (32,20)	2 (22.2)	15 (36.6)	3 (42.9)	2 (100.0)	1 (12.5)	1 (100.0)	4 (21.1)	8.816	0.141 ^a^	--
Obligations	9 (10,30)	0 (0.0)	4 (9.8)	1 (14.3)	1 (50.0)	0 (0.0)	0 (0.0)	3 (15.8)	5.977	0.389 ^a^	--
Situations	12 (13,80)	1 (11.1)	5 (12.2)	2 (28.6)	2 (100.0)	0 (0.0)	0 (0.0)	2 (10.5)	10.439	0.061 ^a^	--
Craving	19 (21,80)	1 (11.1)	9 (22.0)	4 (57.1)	2 (100.0)	1 (12.5)	0 (0.0)	2 (10.5)	11.957	**0.032** ^a^	0.415

Values are expressed as median and IQR (M [IQR]) for continuous variables or as number and percentage (*n* (%)) for categorical variables. ^a^ The exact Fisher test was used to evaluate the difference between the presence of, FA severity and FA’s symptoms in the different EDs. Statistical significance for *p* < 0.05 (in bold). A-AN, atypical anorexia nervosa; ARFID, avoidant restrictive food intake disorder; BED, binge eating disorder; BN, bulimia nervosa; BP-AN, binge purging anorexia nervosa; ED-NOS, eating disorder not otherwise specified; EDs, eating disorders, R-AN, restrictive anorexia nervosa; YFAS, Yale food addiction scale.

**Table 3:** Adjusted association between psychometric scales and Food Addiction.

Dependent Variable	Independent Variables	Coefficient (B)	95% CI		*p*	Exp (B)
			Lower Bound	Upper Bound		
Food addiction	Age	**0.430**	**1.034**	2.288	**0.034**	1.538
	EAT-26	0.046	1.009	1.087	**0.015**	1.047
	Interoceptive Deficits (EDI-3)	0.095	1.024	1.181	**0.009**	1.100
	Constant	−9.662			**0.005**	0.000

Multivariable logistic regressions between Food Addiction (dependent variable) and psychometric scales (independent co-variables). Analyses were adjusted for patients’ age. A separate quantile regression analysis was conducted for each psychometric scale and the final multivariable models were determined through a backward approach. Statistical significance for *p* < 0.05 (in bold). Variables included in the model: Age, Total score (CDI 2), Emotional problems (CDI 2), Social Problem (CDI 2), Total Score (MASC 2), Physical Symptoms (MASC 2), Social Anxiety (MASC 2), EAT-26, Drive for Thinness (EDI-3), Bulimia (EDI-3), Body Dissatisfaction (EDI-3), Low self-Esteem (EDI-3), Personal Alienation (EDI-3), Interpersonal Insecurity (EDI-3), Interpersonal Alienation (EDI-3), Interoceptive Deficits (EDI-3), Emotional Dysregulation (EDI-3), Perfectionism (EDI-3), Ascetism (EDI-3), Maturity Fears (EDI-3), Eating Disorder Risk (EDI-3), EDI-3 Ineffectiveness, Interpersonal Problems (EDI-3), Affective Problems (EDI-3), Overcontrol (EDI-3), General Psychological Maladjustment (EDI-3). The table shows only the final step of the regression. Statistical significance for *p* < 0.05. EAT-26, eating attitude test 26; EDI-3, eating disorders inventory 3; CDI 2, child depression inventory 2; MASC 2, multidimensional anxiety scale for children.

## Supplementary Materials

The following are available online at https://www.mdpi.com/2072-6643/12/5/1524/s1, Table S1: Psychometric tests’ scores for patients with and without FA, Table S2: Psychometric tests’ scores for patients with mild, moderate and severe FA, Table S3. Psychometric tests’ scores included in the multivariable logistic regression for patients with/without FA.

## Abbreviation

A-AN	Atypical Anorexia Nervosa
ARFID	Avoidant Restrictive Food Intake Disorder
BED	Binge Eating Disorder
BMI	Body Mass Index
BN	Bulimia Nervosa
BP-AN	Binge Purging Anorexia Nervosa
CDI 2	Child Depression Inventory 2
DSM–5	Diagnostic and Statistical Manual of Mental Disorders 5th Edition
DSM–IV	Diagnostic and Statistical Manual of Mental Disorders 4th Edition
EAT-26	Eating Attitude Test 26
EDI-3	Eating Disorders Inventory 3
ED-NOS	Eating Disorder Not Otherwise Specified
EDs	Eating Disorders,
IQR	Interquartile Range
MASC 2	Multidimensional Anxiety Scale for Children.
R-AN	Restrictive Anorexia Nervosa
SRAD	Substance-Related and Addictive Disorders
YFAS	Yale Food Addiction Scale
