# Supplementary material for: Food Addiction in a Group of Italian Adolescents Diagnosed for Eating Disorder
Source: Nutrients. 2020 May 23;12(5):1524. doi: 10.3390/nu12051524 (PMC7285060; doi:10.3390/nu12051524)
Supplement: Supplementary file 1 [file nutrients-12-01524-s001.pdf]

Table S1. Psychometric tests' scores for patients with and without FA.

	FA absent				FA present				p^a	Mann-Whitney U	p^b
	**n**	**Mean**	**Median**	**IQR**	**n**	**Mean**	**Median**	**IQR**			
**Activities (YSR)**	35	38.20	38.00	10.00	34	39.59	40.00	14.00	**0.016**	637.00	0.614
**Social (YSR)**	35	38.80	41.00	19.00	33	41.45	43.00	15.00	**0.047**	645.50	0.403
**Academic Performance (YSR)**	6	7.00	2.50	9.00	2	21.50	21.50	0.00	**0.001**	7.50	0.643
**Total Competence (YSR)**	35	36.57	35.00	14.00	33	38.24	37.00	16.00	**0.042**	638.50	0.454
**Anxious/Depressed (YSR)**	35	60.29	59.00	13.00	34	67.56	65.50	15.00	**0.001**	873.00	**0.001**
**Withdrawn/Depressed (YSR)**	35	59.97	58.00	13.00	34	66.82	66.00	16.00	**0.000**	838.00	**0.003**
**Somatic Complaints (YSR)**	35	55.69	52.00	10.00	34	61.09	61.50	16.00	**0.000**	793.50	**0.017**
**Social Problems (YSR)**	35	56.31	55.00	11.00	34	60.18	58.00	11.00	**0.000**	769.50	**0.035**
**Thought Problems (YSR)**	34	54.91	52.00	6.00	34	61.38	61.00	10.00	**0.000**	871.00	**0.000**
**Attention Problems (YSR)**	35	55.34	52.00	7.00	34	57.56	54.00	11.00	**0.000**	769.00	**0.035**
**Rule-Breaking Behaviour (YSR)**	35	51.89	51.00	2.00	34	53.68	51.00	4.00	**0.000**	625.50	0.703
**Aggressive Behaviour (YSR)**	35	53.89	51.00	7.00	34	56.38	57.00	8.00	**0.000**	763.00	**0.041**
**Internalizing Problems (YSR)**	44	56.27	54.00	12.00	43	66.19	67.00	12.00	0.201	1432.50	**0.000**
**Externalizing Problems (YSR)**	44	49.66	51.00	12.00	42	53.55	54.00	10.00	0.490	1163.50	**0.038**
**Total Problems (YSR)**	44	52.11	51.00	13.00	43	60.28	63.00	10.00	0.067	1394.50	**0.000**
**Affective Problems (YSR)**	44	58.73	55.00	12.00	43	67.47	67.00	16.00	**0.000**	1447.50	**0.000**
**Anxiety Problems (YSR)**	44	57.02	52.00	12.00	43	60.65	63.00	13.00	**0.000**	1240.50	**0.012**
**Somatic Problems (YSR)**	44	56.25	54.50	9.00	43	62.12	63.00	16.00	**0.000**	1247.00	**0.010**
**ADHD Problems (YSR)**	44	53.75	51.00	7.00	43	54.91	52.00	6.00	**0.000**	1169.00	0.052
**Oppositional Defiant Problem (YSR)**	44	55.20	52.00	4.00	43	57.12	55.00	8.00	**0.000**	1245.50	**0.010**
**Conduct Problems (YSR)**	44	63.11	50.00	3.00	43	52.51	50.00	3.00	**0.000**	945.00	0.993
**Obsessive-Compulsive Problems (YSR)**	44	56.84	52.00	9.00	43	65.67	66.00	15.00	**0.000**	1475.00	**0.000**
**Post-traumatic Stress Problems (YSR)**	44	57.55	54.00	10.00	43	65.44	66.00	12.00	**0.001**	1461.00	**0.000**
**Positive Qualities (YSR)**	10	50.10	51.00	20.00	20	42.65	44.00	17.00	0.300	56.50	0.055
**Total score (CDI 2)**	44	12.11	9.00	12.00	43	20.98	20.00	10.00	**0.007**	1449.00	**0.000**
**Emotional Problems (CDI 2)**	44	5.64	5.00	6.00	43	11.49	12.00	7.00	**0.001**	1500.50	**0.000**
**Interpersonal Problems (CDI 2)**	44	2.50	2.00	5.00	43	3.49	3.00	3.00	**0.000**	1189.00	**0.037**
**Total Problems (MASC 2)**	44	50.64	49.00	17.00	43	59.21	58.00	20.00	0.141	1287.50	**0.004**
**Physical symptoms (MASC 2)**	44	51.05	48.00	16.00	43	62.65	62.00	17.00	**0.004**	1448.50	**0.000**
**Social anxiety (MASC 2)**	44	52.23	49.00	16.00	43	58.65	61.00	23.00	**0.005**	1232.50	**0.015**

**Separation (MASC 2)**	44	53.45	52.50	18.00	43	54.56	52.00	24.00	**0.001**	967.00	0.858
**Avoidance (MASC 2)**	44	47.27	49.00	14.00	42	48.02	47.00	16.00	0.884	968.50	0.700
**EAT-26**	42	23.12	19.00	26.00	40	43.63	41.50	29.00	**0.039**	1343.00	**0.000**
**BUT (mean)**	42	3.75	1.35	2.56	40	2.95	3.02	1.75	**0.000**	1293.00	**0.000**
**Drive for Thinness (EDI-3)**	42	11.43	8.00	17.00	39	21.00	23.00	11.00	**0.000**	1275.00	**0.000**
**Bulimia (EDI-3)**	42	2.69	0.05	3.00	39	7.00	4.00	7.00	**0.000**	1197.00	**0.000**
**Body Dissatisfaction (EDI-3)**	42	17.07	17.00	20.00	39	27.05	27.00	10.00	**0.013**	2010.50	**0.000**
**Low Self-Esteem (EDI-3)**	42	7.90	6.50	11.00	40	13.73	16.00	9.00	**0.003**	1236.50	**0.000**
**Personal Alienation (EDI-3)**	42	7.12	4.50	9.00	40	13.53	15.50	11.00	**0.001**	1272.00	**0.000**
**Interpersonal Insecurity (EDI-3)**	42	9.19	10.50	11.00	40	13.78	13.50	8.00	0.064	1157.50	**0.003**
**Interpersonal Alienation (EDI-3)**	42	7.38	6.00	9.00	40	11.75	12.50	8.00	**0.007**	1182.00	**0.001**
**Interoceptive Deficits (EDI-3)**	42	9.00	5.00	14.00	40	19.30	20.00	14.00	**0.001**	1360.50	**0.000**
**Emotional Dysregulation (EDI-3)**	42	5.00	2.00	9.00	40	11.85	12.50	8.00	**0.000**	1350.50	**0.000**
**Perfectionism (EDI-3)**	42	6.33	6.00	6.00	40	10.78	10.50	11.00	**0.003**	1205.50	**0.001**
**Ascetism (EDI-3)**	42	6.24	5.00	10.00	40	12.25	12.00	13.00	**0.000**	1255.00	**0.000**
**Maturity Fears (EDI-3)**	42	12.50	11.50	9.00	40	16.53	17.50	13.00	**0.006**	1107.00	**0.013**
**Eating Disorder Risk (EDI-3)**	42	31.19	26.50	42.00	40	55.48	57.00	28.00	**0.004**	1321.00	**0.000**
**Ineffectiveness (EDI-3)**	42	15.02	10.50	19.00	40	27.13	29.00	17.00	**0.003**	1270.50	**0.000**
**Interpersonal Problems (EDI-3)**	42	17.52	16.00	22.00	40	25.53	25.50	17.00	**0.003**	1148.00	**0.004**
**Affective Problems (EDI-3)**	42	14.00	8.00	20.00	40	31.15	32.00	22.00	**0.003**	1374.00	**0.000**
**Overcontrol (EDI-3)**	42	12.57	11.00	17.00	40	23.03	21.50	21.00	**0.006**	1271.50	**0.000**
**General Psychological Maladjustment (EDI-3)**	41	70.73	52.00	79.00	40	123.35	125.00	49.00	**0.001**	1303.00	**0.000**

Values are expressed as mean, median and IQR (M [IQR]).

[a] The Shapiro-Wilk test was performed to evaluate variables distribution. Variables are considered non-normally distributed for $p<0.05$ (in bold).

[b] The Mann-Whitney U test was performed to compare psychometrics tests' scores between the two groups of FA (absent vs present). Statistical significance for $p<0.05$ (in bold).

EAT-26, eating attitude test 26; EDI-3, eating disorders inventory 3; CDI 2, child depression inventory 2; FA, Food Addiction; IQR, Interquartile Range; MASC 2, multidimensional anxiety scale for children.

Table S2. Psychometric tests' scores for patients with mild, moderate and severe FA.

	FA mild				FA moderate				FA severe				Kruskal-Wallis	p[a]
	n	Mean	Median	IQR	n	Mean	Median	IQR	n	Mean	Median	IQR		
**Activities (YSR)**	10	38.80	39.00	15.75	10	40.70	40.50	11.00	14	39.36	36.50	16.00	0.614	0.736
**Social (YSR)**	9	46.33	47.00	20.50	10	39.40	40.00	16.75	14	39.79	39.50	12.00	2.973	0.226
**Total Competence (YSR)**	10	41.20	38.50	23.00	10	37.20	37.50	11.00	13	36.77	37.00	15.00	0.765	0.682
**Anxious/Depressed (YSR)**	10	63.80	64.00	14.25	10	70.00	66.50	13.00	14	68.50	67.50	20.00	1.653	0.438
**Withdrawn/Depressed (YSR)**	10	61.10	58.00	14.75	10	69.20	67.50	17.00	14	69.21	67.50	24.00	4.243	0.120
**Somatic Complaints (YSR)**	10	64.90	65.00	16.00	10	59.00	61.50	27.50	14	59.86	60.00	16.00	0.356	0.837
**Social Problems (YSR)**	10	56.90	55.00	10.50	10	62.90	64.00	10.00	14	60.57	59.50	10.00	3.359	0.187
**Thought Problems (YSR)**	10	58.60	55.00	17.75	10	62.60	63.50	8.75	14	62.50	59.50	11.00	2.160	0.340
**Attention Problems (YSR)**	10	54.90	52.00	9.25	10	58.30	60.00	9.25	14	58.93	55.50	12.00	3.157	0.206
**Rule-Breaking Behaviour (YSR)**	10	53.20	51.00	5.25	10	54.30	50.00	7.75	14	53.57	51.00	4.00	0.350	0.839
**Aggressive Behaviour (YSR)**	10	57.40	59.50	8.50	10	56.60	57.50	12.00	14	55.50	55.00	9.00	0.938	0.626
**Internalizing Problems (YSR)**	15	63.60	67.00	19.00	12	69.42	68.00	15.00	16	66.19	66.50	10.00	0.902	0.637
**Externalizing Problems (YSR)**	14	53.64	56.00	10.00	12	55.08	56.50	13.50	16	52.31	53.00	7.00	1.653	0.438
**Total Problems (YSR)**	15	57.20	57.00	17.00	12	63.75	65.00	4.25	16	60.56	62.50	9.00	2.797	0.247
**Affective Problems (YSR)**	15	65.33	63.00	16.00	12	68.67	67.00	6.00	16	68.56	68.00	17.00	0.801	0.670
**Anxiety Problems (YSR)**	15	59.47	63.00	11.00	12	63.75	65.50	13.50	16	59.44	59.00	16.00	2.838	0.242
**Somatic Problems (YSR)**	15	62.47	60.00	13.00	12	65.75	63.00	16.75	16	59.06	56.50	14.00	3.137	0.208
**ADHD Problems (YSR)**	15	53.07	52.00	4.00	12	57.92	58.50	7.50	16	54.38	52.00	3.00	5.301	0.071
**Oppositional Defiant Problem (YSR)**	15	56.33	55.00	8.00	12	58.33	57.50	11.25	16	56.94	55.00	12.00	1.139	0.566
**Conduct Problems (YSR)**	15	52.00	51.00	1.00	12	52.92	50.50	3.00	16	52.69	50.00	5.00	0.530	0.767
**Obsessive-Compulsive Problems (YSR)**	15	61.87	63.00	21.00	12	68.00	67.00	11.50	16	67.50	66.00	24.00	2.230	0.328
**Post-traumatic Stress Problems (YSR)**	15	61.27	63.00	17.00	12	69.42	68.50	11.25	16	66.38	66.00	10.00	4.876	0.087
**Positive Qualities (YSR)**	3	43.00	44.00	0.00	5	38.80	35.00	16.50	12	44.17	45.00	19.00	1.349	0.509
**Total score (CDI 2)**	15	18.47	19.00	19.00	12	23.25	21.00	8.00	16	21.63	20.00	9.00	1.788	0.409
**Emotional Problems (CDI 2)**	15	10.60	10.00	10.00	12	12.83	12.00	6.25	16	11.31	12.00	8.00	0.962	0.618
**Interpersonal Problems (CDI 2)**	15	3.07	3.00	4.00	12	4.33	4.50	4.25	16	3.25	3.00	3.00	2.170	0.338
**Total Problems (MASC 2)**	15	55.47	57.00	28.00	12	65.67	67.00	18.75	16	57.88	56.00	25.00	2.297	0.317
**Physical symptoms (MASC 2)**	15	61.93	56.00	30.00	12	66.00	67.00	19.00	16	60.81	61.00	16.00	1.279	0.528
**Social anxiety (MASC 2)**	15	55.80	61.00	32.00	12	62.08	60.00	22.00	16	58.75	60.00	20.00	1.670	0.434
**Separation (MASC 2)**	15	49.53	45.00	17.00	12	57.83	62.50	23.25	16	56.81	53.00	28.00	3.403	0.182
**Avoidance (MASC 2)**	14	44.07	44.00	25.50	12	53.17	53.00	14.50	16	47.63	48.00	13.00	3.293	0.193

**EAT-26**	14	38.57	30.00	32.00	11	43.45	49.00	25.00	15	48.47	49.00	26.00	2.396	0.302
**BUT (mean)**	14	2.66	2.92	2.19	11	3.10	3.00	1.20	15	3.13	3.11	1.98	0.627	0.731
**Drive for Thinness (EDI-3)**	13	17.08	19.00	18.00	11	23.09	23.00	7.00	15	22.87	25.00	9.00	3.038	0.219
**Bulimia (EDI-3)**	13	4.31	3.00	7.00	11	4.91	4.00	7.00	15	10.87	8.00	11.00	5.901	0.052
**Body Dissatisfaction (EDI-3)**	13	25.46	26.00	10.50	11	28.27	27.00	13.00	15	27.53	28.00	10.00	0.837	0.658
**Low Self-Esteem (EDI-3)**	14	13.14	16.00	11.75	11	13.64	16.00	9.00	15	14.33	14.00	8.00	0.020	0.990
**Personal Alienation (EDI-3)**	14	12.86	14.50	12.25	11	14.55	16.00	13.00	15	13.40	15.00	10.00	0.259	0.879
**Interpersonal Insecurity (EDI-3)**	14	12.57	11.00	13.50	11	13.27	12.00	10.00	15	15.27	15.00	6.00	1.687	0.430
**Interpersonal Alienation (EDI-3)**	14	10.64	11.50	11.75	11	11.64	11.00	7.00	15	12.87	14.00	6.00	0.475	0.788
**Interoceptive Deficits (EDI-3)**	14	16.36	14.50	11.75	11	21.55	23.00	10.00	15	20.40	24.00	21.00	2.466	0.291
**Emotional Dysregulation (EDI-3)**	14	11.57	11.50	10.75	11	11.91	13.00	7.00	15	12.07	12.00	10.00	0.029	0.985
**Perfectionism (EDI-3)**	14	8.71	7.50	8.50	11	11.36	11.00	8.00	15	12.27	12.00	8.00	2.595	0.273
**Ascetism (EDI-3)**	14	10.29	9.00	11.25	11	13.45	12.00	12.00	15	13.20	12.00	17.00	1.514	0.469
**Maturity Fears (EDI-3)**	14	15.50	13.50	10.75	11	16.09	16.00	15.00	15	17.80	19.00	9.00	0.951	0.622
**Eating Disorder Risk (EDI-3)**	14	48.79	49.50	38.50	11	56.27	56.00	13.00	15	61.13	61.00	22.00	2.604	0.272
**Ineffectiveness (EDI-3)**	14	25.64	30.50	23.00	11	28.18	29.00	19.00	15	27.73	27.00	14.00	0.033	0.983
**Interpersonal Problems (EDI-3)**	14	23.21	24.50	22.00	11	24.91	25.00	17.00	15	28.13	26.00	14.00	1.047	0.592
**Affective Problems (EDI-3)**	14	27.93	28.00	21.00	11	33.45	33.00	17.00	15	32.47	33.00	27.00	1.180	0.554
**Overcontrol (EDI-3)**	14	19.00	18.00	13.75	11	24.82	25.00	19.00	15	25.47	26.00	23.00	2.441	0.295
**General Psychological Maladjustment (EDI-3)**	14	111.29	124.50	77.25	11	127.45	124.00	73.00	15	131.60	127.00	36.00	1.103	0.576

Values are expressed as mean, median and IQR (M [IQR]).

[a] The Kruskal-Wallis test was performed to compare psychometrics tests' scores between the three group of severity of FA (mild, moderate, severe). Statistical significance for p<0.05 (in bold).

The Academic Performance scale of the YSR questionnaire was not included due to the groups size (n=0 for mild FA, n=1 for moderate and severe FA)

EAT-26, eating attitude test 26; EDI-3, eating disorders inventory 3; CDI 2, child depression inventory 2; FA, Food Addiction; IQR, Interquartile Range; MASC 2, multidimensional anxiety scale for children.

Table S3. Psychometric tests' scores included in the multivariable logistic regression for patients with/without FA.

	FA absent (n=41)			FA present (n=38)			B[a]	95% CI[a]		P[a]	Exp(B)[a]
	Mean	Median	IQR	Mean	Median	IQR		Lower Bound	Upper Bound		
**Internalizing Problems (YSR)**	56.70	54.00	12.00	66.39	67.00	12.00	0.083	1.039	1.136	**0.000**	1.087
**Externalizing Problems (YSR)**	49.20	51.00	12.00	53.71	54.00	10.00	0.066	1.007	1.132	**0.027**	1.068
**Total Problems (YSR)**	52.00	51.00	14.00	60.61	63.00	9.00	0.094	1.043	1.156	**0.000**	1.098
**Total score (CDI 2)**	12.20	9.00	14.00	21.58	20.50	10.00	0.108	1.055	1.176	**0.000**	1.114
**Emotional Problems (CDI 2)**	5.78	5.00	7.00	11.66	12.00	7.00	0.214	1.122	1.368	**0.000**	1.239
**Interpersonal Problems (CDI 2)**	2.37	2.00	4.00	3.61	3.00	3.00	0.158	0.985	1.393	0.074	1.171
**Total score (MASC 2)**	51.20	49.00	18.00	60.21	59.00	25.00	0.043	1.011	1.079	**0.009**	1.044
**Physical symptoms (MASC 2)**	51.60	48.00	16.00	62.68	66.00	18.00	0.084	1.042	1.135	**0.000**	1.088
**Social anxiety (MASC 2)**	52.50	49.00	16.00	60.00	62.00	22.00	0.043	1.007	1.082	**0.020**	1.044
**EAT-26**	22.60	19.00	26.00	43.21	41.50	28.00	0.066	1.036	1.102	**0.000**	1.068
**Drive for Thinness (EDI-3)**	11.20	8.00	18.00	21.18	23.00	10.00	0.116	1.061	1.188	**0.000**	1.123
**Bulimia (EDI-3)**	2.63	0.00	2.00	7.18	4.00	7.00	0.141	1.043	1.271	**0.005**	1.151
**Body Dissatisfaction (EDI-3)**	16.50	17.00	19.00	27.00	27.00	10.00	0.096	1.046	1.159	**0.000**	1.101
**Low Self-Esteem (EDI-3)**	8.07	7.00	11.00	13.84	16.00	9.00	0.130	1.059	1.224	**0.000**	1.138
**Personal Alienation (EDI-3)**	7.27	5.00	9.00	13.45	15.50	12.00	0.140	1.069	1.238	**0.000**	1.150
**Interpersonal Insecurity (EDI-3)**	9.27	11.00	11.00	13.66	13.50	9.00	0.112	1.039	1.205	**0.003**	1.119
**Interpersonal Alienation (EDI-3)**	7.44	6.00	9.00	11.39	11.5	8.00	0.122	1.043	1.223	**0.003**	1.129
**Interoceptive Deficits (EDI-3)**	8.80	5.00	14.00	19.45	20.00	14.00	0.128	1.072	1.205	**0.000**	1.136
**Emotional Dysregulation (EDI-3)**	4.98	2.00	9.00	11.76	12.50	8.00	0.188	1.107	1.316	**0.000**	1.207
**Perfectionism (EDI-3)**	6.07	6.00	7.00	10.58	10.50	11.00	0.143	1.056	1.260	**0.002**	1.153
**Ascetism (EDI-3)**	6.20	5.00	11.00	12.18	12.00	13.00	0.132	1.060	1.227	**0.000**	1.141
**Maturity Fears (EDI-3)**	12.60	12.00	9.00	16.79	18.00	14.00	0.091	1.022	1.174	**0.010**	1.095
**Eating Disorder Risk (EDI-3)**	30.40	26.00	42.00	55.32	57.00	27.00	0.052	1.028	1.080	**0.000**	1.053
**Ineffectiveness (EDI-3)**	15.30	11.00	19.00	27.16	29.50	18.00	0.077	1.038	1.124	**0.000**	1.080
**Interpersonal Problems (EDI-3)**	17.70	17.00	23.00	25.05	25.00	16.00	0.053	1.015	1.096	**0.006**	1.055
**Affective Problems (EDI-3)**	13.80	8.00	20.00	31.21	32.00	22.00	0.093	1.053	1.143	**0.000**	1.097
**Overcontrol (EDI-3)**	12.30	11.00	17.00	22.76	21.50	44.00	0.086	1.041	1.141	**0.000**	1.090

**General Psychological Maladjustment (EDI-3)**	70.70	52.00	79.00	122.97	125.00	53.00	0.026	1.014	1.038	**0.000**	1.026

Values are expressed as mean, median and IQR (M [IQR])

[a] Univariable logistic regression was performed to investigate the association between the presence of FA (dependent variable) and psychometric scores (independent variables). Statistical significance for p<0.05 (in bold).

EAT-26, eating attitude test 26; EDI-3, eating disorders inventory 3; CDI 2, child depression inventory 2; CI, confidence interval; IQR, interquartile range; MASC 2, multidimensional anxiety scale for children.
